# Supplementary material for: Leucine aminopeptidase1 controls egg deposition and hatchability in male Aedes aegypti mosquitoes
Source: Nat Commun. 2024 Jan 2;15:106. doi: 10.1038/s41467-023-44444-z (PMC10762072; doi:10.1038/s41467-023-44444-z)
Supplement: Supplementary file 1 — Supplementary Information [file 41467_2023_44444_MOESM1_ESM.pdf]

Supplementary Information

Leucine aminopeptidase1 controls egg deposition and hatchability in male *Aedes aegypti* mosquitoes

Sun et al

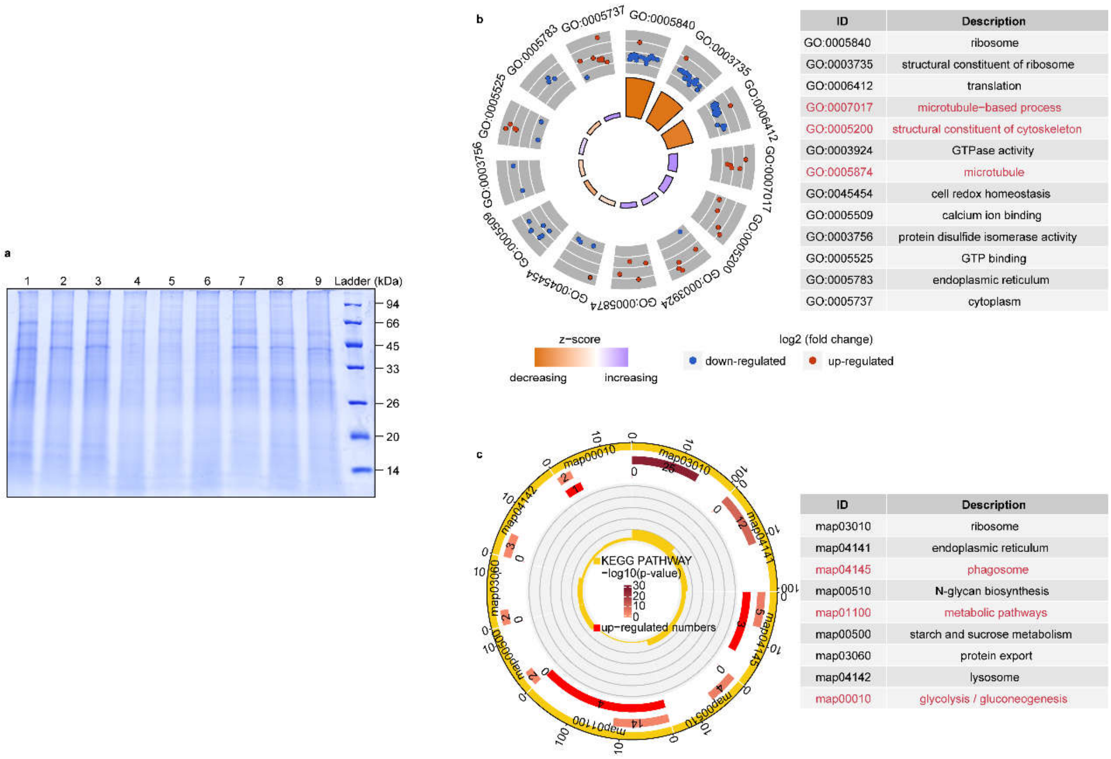

Supplementary Fig. 1 Functional classification of DEPs. a SDS–PAGE gel showing the protein profile of extracts at G1 (lane 1, 2, and 3), G2 (lane 4, 5, and 6), G3 (lane 7, 8, and 9). Each sample was repeated for three times. **b** GO enrichment analysis. The graph displays the enrichment results in a circular format. From outside to inside: GO terms, the protein changes (up- and down- regulated proteins are marked with red and blue spots), and the z-score. The descriptions of the corresponding GO terms are shown on the right, and GO clusters including the completely up-regulated proteins are labelled with red. The DAVID web server was used for GO enrichment analysis and the *p*-value was determined by a two-sided Fisher's exact test with Benjamini-Hochberg adjustment. **c** KEGG functional classification. From outside to inside: KEGG terms, *p*-value (numbers and colors represent numbers of enriched proteins in the term and the *p*-value, respectively), the numbers of up-regulated proteins in the term, and rich factor (numbers of enriched proteins/total proteins in the term). The descriptions of the corresponding KEGG terms are shown on the right, and KEGG clusters enriched to the up-regulated proteins are labelled with red. The KOBAS web server was used for KEGG enrichment analysis and the *p*-value was determined by a two-sided Fisher's exact test with Benjamini-Hochberg adjustment. Source data are provided as a Source

Data file.

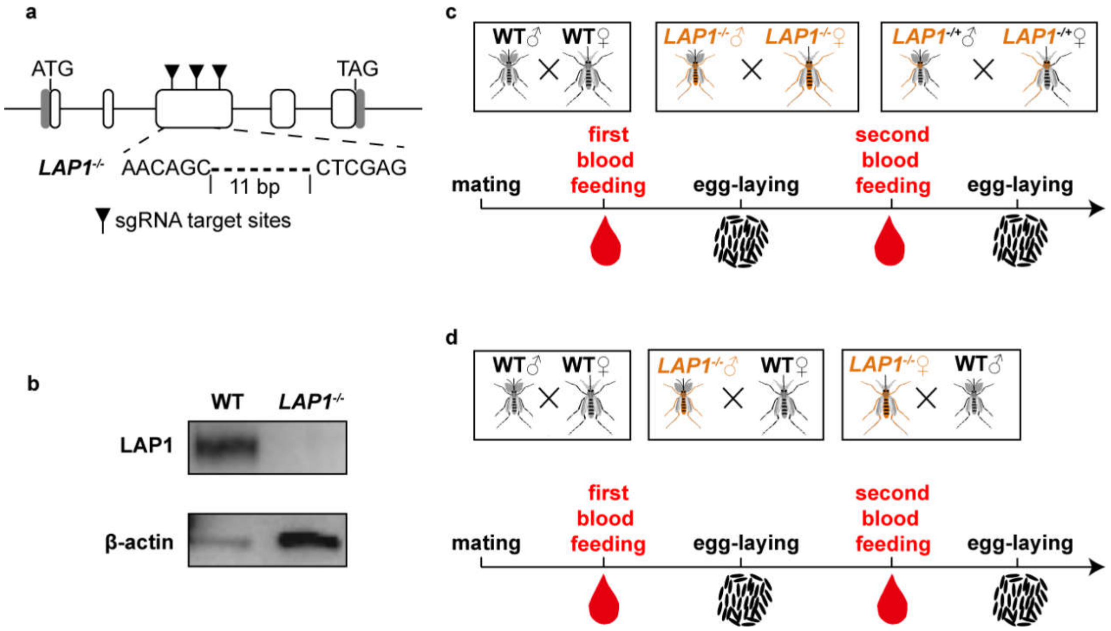

Supplementary Fig. 2 Generation of the *LAP1* knock-out mutant. a Schematic representation of the CRISPR/Cas9-mediated genome editing of *LAP1*. The location of the exon, sgRNA target sites, and missing base are shown. **b** The protein levels of LAP1 in WT and *LAP1*$^{-/-}$ male mosquitoes were quantified by Western blot with mouse anti-LAP1 antibody. β-Actin levels were used as loading control. The experiments were repeated three times with similar results. **c** Schematic representation of the mating study (WT♂ × WT♀, *LAP1*$^{-/-}$♂ × *LAP1*$^{-/-}$♀, and *LAP1*$^{-/+}$♂ × *LAP1*$^{-/+}$♀). **d** Schematic representation of the mating study (WT♂ × WT♀, *LAP1*$^{-/-}$♂ × WT♀, and WT♂ × *LAP1*$^{-/-}$♀). Source data are provided as a Source Data file.

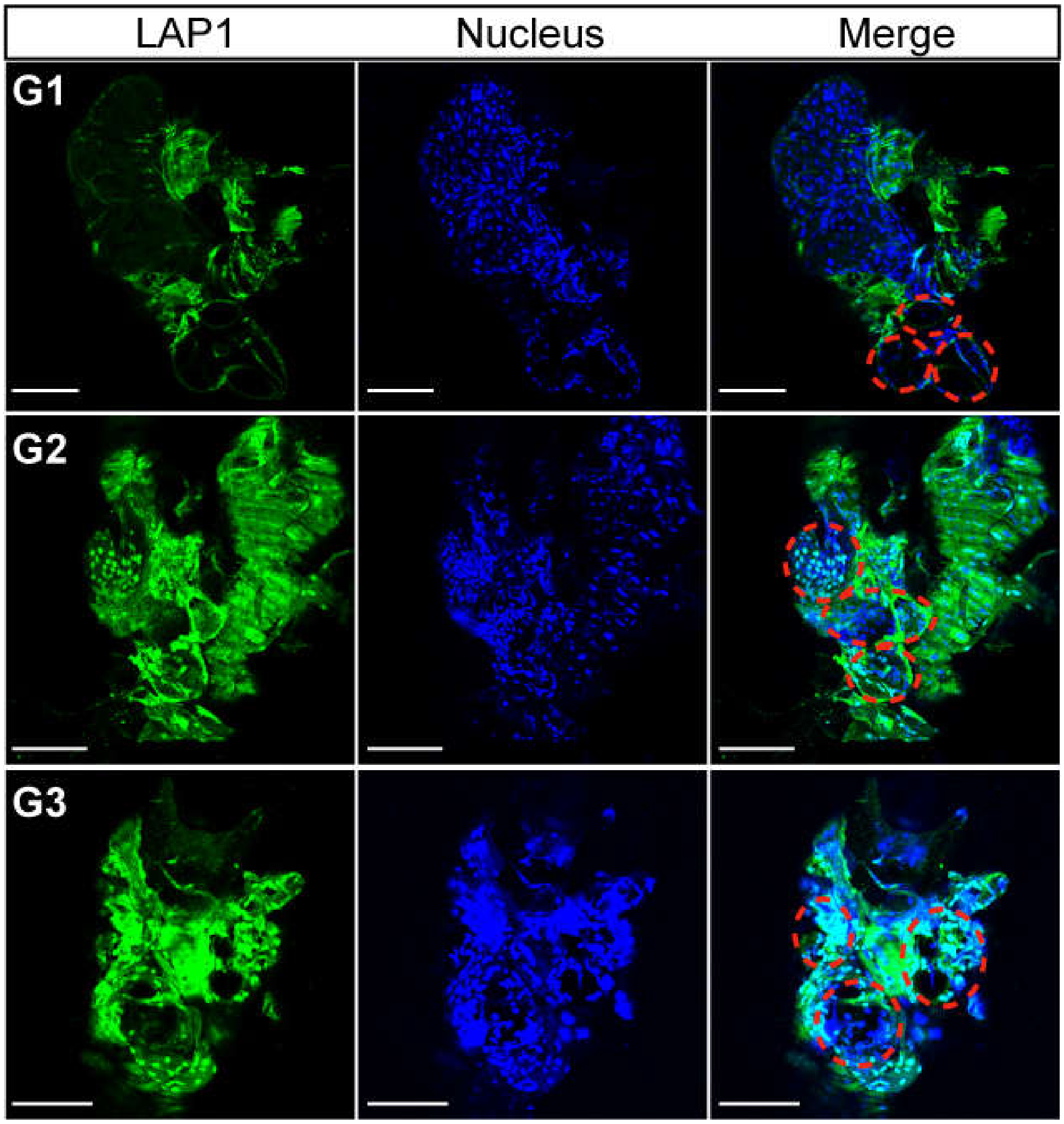

Supplementary Fig. 3 The distribution of *LAP1* in the *Ae. aegypti* spermathecae. Detection of LAP1 protein in G1, G2, and G3 *Ae. aegypti* spermathecae by immunofluorescence assay with mouse anti-LAP1 antibody (green). Hoechst 33258 (blue) was used to stain the nucleus. Spermathecae are marked with red dashed circles. Scale bar: 100 μm. The experiments were repeated three times with similar results. Source data are provided as a Source Data file.

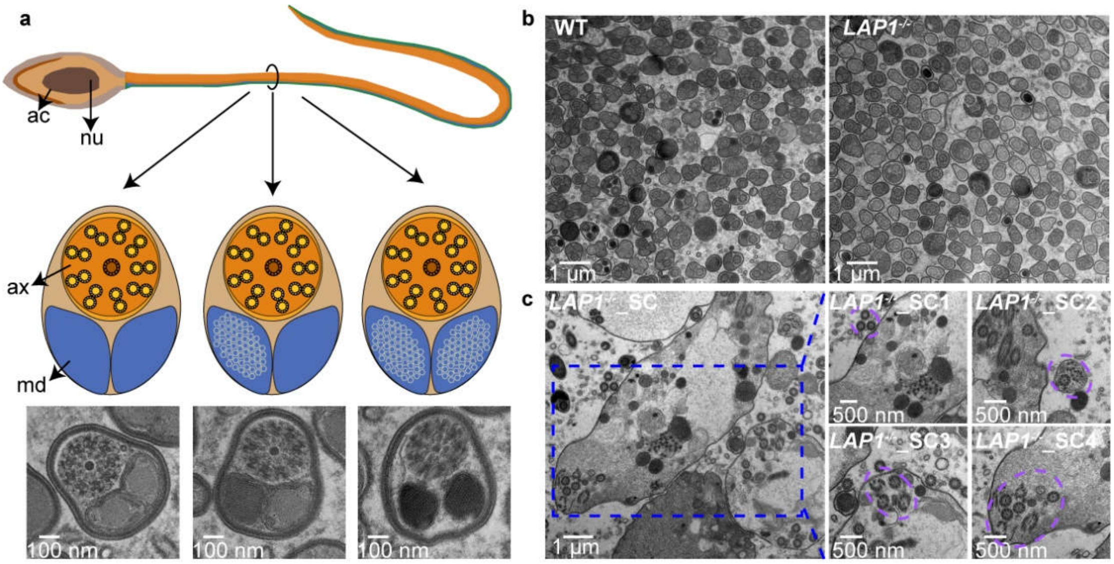

Supplementary Fig. 4 TEM analysis of the spermatozoa structure in WT and *LAP1*$^{-/-}$ mosquitoes. a Schematic structure of the mosquito sperm. ac: acrosome; nu: nucleus; ax: axoneme; md: mitochondrial derivative. **b** The cross-section of WT and *LAP1*$^{-/-}$ spermatozoa detected by TEM. Scale bars: 1 μm. **c** Detection of mitochondrial autophagy by TEM. Autophagosomes in *LAP1*$^{-/-}$ spermatozoa were labelled with blue dotted box. Purple ellipse dashed line: disintegrated sperm. The experiments were repeated three times with similar results. Source data are provided as a Source Data file.

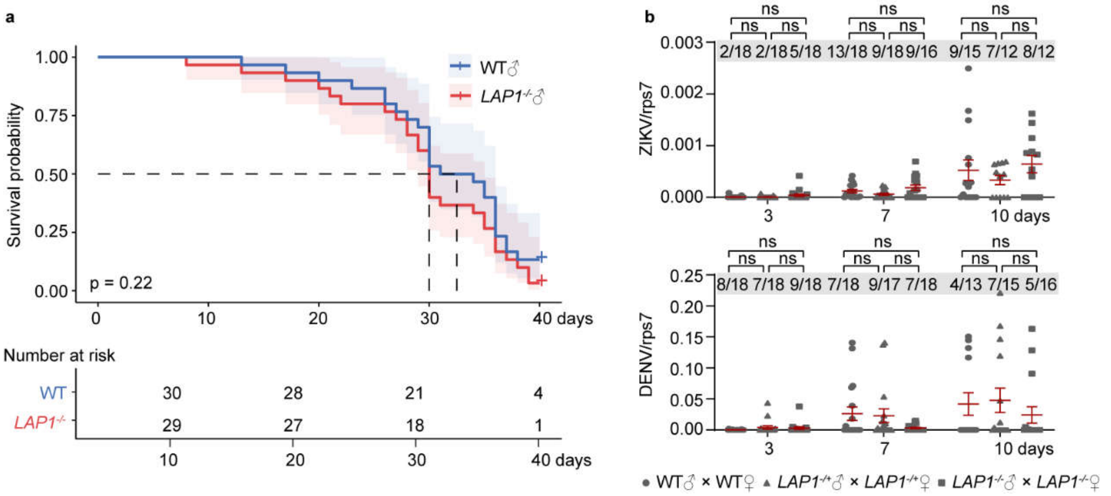

Supplementary Fig. 5 The assessment of physiological indicators and safety of *LAP1*$^{-/-}$ males. a The survival rate of WT and *LAP1*$^{-/-}$ males (n = 30). Survival curves were compared using the log-rank test (Mantel-Cox test). **b** Transmissibility of virus in *LAP1*$^{-/-}$ mosquitoes. Seventy female mosquitoes in *LAP1*$^{-/-}$♂ × *LAP1*$^{-/-}$♀, *LAP1*$^{-/+}$♂ ×

LAP1$^{-/+}$♀, and WT♂ × WT♀ groups were infected with ZIKV (For WT♂ × WT♀, n = 18 for both 3 and 7 days; n = 15 for 10 days. For *LAP1*$^{-/+}$♂ × *LAP1*$^{-/+}$♀, n = 18 for both 3 and 7 days; n = 12 for 10 days. For *LAP1*$^{-/-}$♂ × *LAP1*$^{-/-}$♀, n = 18 for 3 days; n = 16 for 7 days; n = 12 for 10 days) or DENV (For WT♂ × WT♀, n = 18 for both 3 and 7 days; n = 13 for 10 days. For *LAP1*$^{-/+}$♂ × *LAP1*$^{-/+}$♀, n = 18 for 3 days; n = 17 for 7 days; n = 15 for 10 days. For *LAP1*$^{-/-}$♂ × *LAP1*$^{-/-}$♀, n = 18 for both 3 and 7 days; n = 16 for 10 days). Total RNA was extracted from a single mosquito at 3-, 7-, and 10-days post infection, and the viral RNA level was measured by qPCR. The top of each column shows the number of infected mosquitoes relative to total mosquitoes. Each dot represents one mosquito. Data are shown as mean ± SEM. Statistical significance was determined using the multiple Mann-Whitney test with Benjamini-Hochberg adjustment. ns: not significant. The experiments were repeated three times with similar results. Source data are provided as a Source Data file.

Supplementary Table 1 List of selected 25 up-regulated proteins in the G2_vs_G1 group for Sankey dot analysis.

**Description**	**Gene Ratio**	***p*- value**	**geneID**	**Count**
microtubule-based process	0.238095238	1.62287E-08	AAEL012424-PB/AAEL012101-PB/AAEL005052-PA/AAEL002851-PA/AAEL011478-PC	5
structural constituent of cytoskeleton	0.19047619	1.05963E-06	AAEL012424-PB/AAEL012101-PB/AAEL005052-PA/AAEL002851-PA	4
cytoplasm	0.333333333	1.03197E-05	AAEL012424-PB/AAEL012101-PB/AAEL010563-PB/AAEL005052-PA/AAEL002851-PA/AAEL006975-PB/AAEL000424-PA	7
microtubule	0.19047619	7.01959E-05	AAEL012424-PB/AAEL012101-PB/AAEL005052-PA/AAEL002851-PA	4
GTPase activity	0.19047619	0.000103859	AAEL012424-PB/AAEL012101-PB/AAEL005052-PA/AAEL002851-PA	4
GTP binding	0.19047619	0.001999811	AAEL012424-PB/AAEL012101-PB/AAEL005052-PA/AAEL002851-PA	4
aminopeptidase activity	0.095238095	0.016737091	AAEL006975-PB/AAEL000424-PA	2
metalloexopeptidase activity	0.095238095	0.016737091	AAEL006975-PB/AAEL000424-PA	2
manganese ion binding	0.095238095	0.023359225	AAEL006975-PB/AAEL000424-PA	2

List of selected 25 up-regulated proteins in the G2_vs_G1 group for Sankey dot analysis.

**Protein**	**Gene description**	**Abbreviations**
AAEL000424-PA	leucine aminopeptidase2	LAP2
AAEL006975-PB	leucine aminopeptidase1	LAP1
AAEL010563-PB	tyrosyl-tRNA synthetase	Ttrs
AAEL011478-PC	cytoplasmic dynein light chain	Cdlc
AAEL002851-PA	Tubulin beta chain1	Tbc1
AAEL005052-PA	Tubulin beta chain2	Tbc2
AAEL012101-PB	tubulin alpha chain1	Tac1
AAEL012424-PB	tubulin alpha chain2	Tac2

Supplementary Table 2 List of LAP1, DBF4, and other 8 candidate proteins

AAEL number	Description	Abbreviations	RNAi efficiency
AAEL008779-PB	DBF4-type zinc finger-containing protein 2 homolog	*DBF4*	-51.30%
AAEL007547-PB	chloride channel protein	*Ccp*	-76.40%
AAEL004231-PA	M12 mutant protein precursor	*M12*	-49.60%
AAEL019957-PA	gamma-glutamyltranspeptidase	*Ggase*	-40.30%
AAEL010738-PC	sodium bicarbonate cotransporter	*Sbc*	-65.80%
AAEL006373-PB	serine protease htra2	*Sph2*	-21.70%
AAEL013528-PA	TPX1: thioredoxin peroxidase	*TPX1*	-60.40%
AAEL019487-PA	growth hormone-inducible transmembrane protein	*Ghitp*	-29.10%
AAEL006975-PB	leucine aminopeptidase1	*LAP1*	-85.70%
AAEL009051-PB	TPX5: Thioredoxin Peroxidase	*TPX5*	-50.90%

Supplementary Table 3 The primers used in this study

Gene name	Forward primers (5'-3')	Reverse primers (5'-3')	Primer use
DBF4	T7-ACCTGTGGTCCAAGATTCGTCA	T7-TCAACACTTTCTGGGTGGGCAT	dsRNA
Ccp	T7-TTTCATTCCGACGTTGTTGGT	T7-TCCGTTATGCGTGGTGTTCTT	dsRNA
M12	T7-AAACCAACGAAAACGCCTACA	T7-GCGATAACACCTCCCCTAAAA	dsRNA
Ggase	T7-AGTACATTCGTTTCCGTTGCC	T7-GTCGTTGAGGATTATTCCCGT	dsRNA
Sbc	T7-ACGATTGCTCGTGTCTACCAC	T7-ATAGCCACAGCCCTTCTTTAA	dsRNA
Sph2	T7-GAAGACTATCAAGCAGGGCGG	T7-GGTATTGTTCAGGGAAAGGGG	dsRNA
TPX1	T7-CGAAAGTAGCAAAGGCCG	T7-TAATGAACAAACCCCGCA	dsRNA
Ghitp	T7-GTACCAGCACCCTGGACAACT	T7-CTCCGCTCGCTTAACAATCTT	dsRNA
LAP1	T7-TGGAGTTGTTCGATTCGCC	T7-CGTCCTGCATTGCTGGTGT	dsRNA
TPX5	T7-GAAATGTGAAGCCGATTGCC	T7-TTCGTTACACCGTTGGGGAA	dsRNA
EGFP	T7-CACAAGTTCAGCGTGTCCG	T7-GTTCACCTTGATGCCGTTC	dsRNA
DBF4	ATCCTTGCAGTGAACCTCGAA	AGGGGGTTGCTGAACTGTGAC	qPCR
Ccp	ACCTATGGCCTGAGCGTTT	GATGTTTCCCGTCGTTTCC	qPCR
M12	ACGTGGCAAGTTCAAAGAGGA	TTGTAGGCGTTTTCGTTGGTT	qPCR
Ggase	CCGATCAACGGGAATAATCCT	GTGAACCACCTGCTCCACCAA	qPCR
Sbc	CTCGTTCAACCATTCCACGAG	AGATGATAGCCACAGCCCTTC	qPCR
Sph2	TGGAGACGAGGTCAATGCG	ACGGTGGGAAACAGCGAAA	qPCR
TPX1	CTCGCTGCGGGGTTTGTT	ATCGGCGTTGGATTTCGG	qPCR
Ghitp	CCAATATGCCCGAGAAGTGA	GTACCGGAGCCAAAACCAAG	qPCR
LAP1	CGGATTTCAGTCGACCCAAT	AACAAGCCTCTCGTCCATGC	qPCR
TPX5	GAGTGGGCTGGAGATTCCTG	GCTTCACATTTCGTTTGGCG	qPCR
DENV	CAGGCTATGGCACTGTCACGAT	CCATTTGCAGCAACACCATCTC	qPCR
ZIKV	GCCATCACTGGGTCTCATCAATAG	CTCGTCTCTTCTTCTCCTTCCTAGC	qPCR
rps7	TCAGTGTACAAGAAGCTGACCGGA	TTCCGCGCGCGCTCACTTATTAGATT	qPCR
LAP1 - sgRNA1	GAAATTAATACGACTCACTATAGAGTTGTTCGATTCGCCGGAGTTTTAGAGCTAGAAATAGC	AAAAGCACCGACTCGGTGCCACTTTTTCAAGTTGATAACGGACTAGCCTTATTTTAACTTGCTATTTCTAGCTCTAAAAC	Knockout
LAP1 - sgRNA2	GAAATTAATACGACTCACTATAGAGCTTGAACAGCTGGACGAGTTTTAGAGCTAGAAATAGC	AAAAGCACCGACTCGGTGCCACTTTTTCAAGTTGATAACGGACTAGCCTTATTTTAACTTGCTATTTCTAGCTCTAAAAC	Knockout

LAP1 -sgRNA3	GAAATTAATACGACTCACTATAGGAATTACGTTCAACAGTGGGTTTTAGAGCTAGAAATAGC	AAAAGCACCGACTCGGTGCCACTTTTTCAAGTTGATAACGGACTAGCCTTATTTTAACTTGCTATTTCTAGCTCTAAAAC	Knockout
LAP1 -knockout-identification	GTCAAATCTGAAAGGAAAAGTTGGA	ATGTTCTCACACAGTGGGATGAGG	PCR
