# Supplementary material for: Leucine aminopeptidase1 controls egg deposition and hatchability in male Aedes aegypti mosquitoes
Source: Nat Commun. 2024 Jan 2;15:106. doi: 10.1038/s41467-023-44444-z (PMC10762072; doi:10.1038/s41467-023-44444-z)
Supplement: Supplementary file 3 — Description of Additional Supplementary Files [file 41467_2023_44444_MOESM3_ESM.pdf]

Description of Additional Supplementary Files

File Name: Supplementary Data 1
Description: Supplementary Data 1 List of identified total proteins in G2_vs_G1, G3_vs_G1, and G3_vs_G2 groups.

File Name: Supplementary Data 2
Description: Supplementary Data 2 List of differentially expressed proteins (DEPs) in G2_vs_G1, G3_vs_G1, and G3_vs_G2 groups.
