# Supplementary material for: Leucine aminopeptidase1 controls egg deposition and hatchability in male Aedes aegypti mosquitoes
Source: Nat Commun. 2024 Jan 2;15:106. doi: 10.1038/s41467-023-44444-z (PMC10762072; doi:10.1038/s41467-023-44444-z)
Supplement: Supplementary file 6 — Reporting Summary [file 41467_2023_44444_MOESM6_ESM.pdf]

nature portfolio

Corresponding author(s): Zhen Zou

Last updated by author(s): Dec 3, 2023

Reporting Summary

Nature Portfolio wishes to improve the reproducibility of the work that we publish. This form provides structure for consistency and transparency in reporting. For further information on Nature Portfolio policies, see our Editorial Policies and the Editorial Policy Checklist.

Statistics

For all statistical analyses, confirm that the following items are present in the figure legend, table legend, main text, or Methods section.

n/a	Confirmed	
☐	☒	The exact sample size (*n*) for each experimental group/condition, given as a discrete number and unit of measurement
☐	☒	A statement on whether measurements were taken from distinct samples or whether the same sample was measured repeatedly
☐	☒	The statistical test(s) used AND whether they are one- or two-sided<br>*Only common tests should be described solely by name; describe more complex techniques in the Methods section.*
☐	☒	A description of all covariates tested
☐	☒	A description of any assumptions or corrections, such as tests of normality and adjustment for multiple comparisons
☐	☒	A full description of the statistical parameters including central tendency (e.g. means) or other basic estimates (e.g. regression coefficient) AND variation (e.g. standard deviation) or associated estimates of uncertainty (e.g. confidence intervals)
☐	☒	For null hypothesis testing, the test statistic (e.g. *F*, *t*, *r*) with confidence intervals, effect sizes, degrees of freedom and *P* value noted<br>*Give P values as exact values whenever suitable.*
☒	☐	For Bayesian analysis, information on the choice of priors and Markov chain Monte Carlo settings
☒	☐	For hierarchical and complex designs, identification of the appropriate level for tests and full reporting of outcomes
☒	☐	Estimates of effect sizes (e.g. Cohen's *d*, Pearson's *r*), indicating how they were calculated

Our web collection on statistics for biologists contains articles on many of the points above.

Software and code

Policy information about availability of computer code

Data collection	MaxQuant; Peakview; Image Lab software (Image Lab 5.2); Zeiss Zen 2010 software (version 6.0); CellSens software (version 1.6)GraphPad Prism statistical software; DAVID web server ; KOBAS web server; Proteomic data were performed using http://www.bioinformatics.com.cn, which is used for analysis and visualization.

Data analysis	Volcano plot analysis in Fig. 1a, GO enrichment analysis in Supplementary Fig. 1b, KEGG functional classification in Supplementary Fig. 1c, and were performed by enhancedvolcano R package (1.13.2), Goplot R package (1.0.2), and circlize R package (0.4.15) in the R environment, respectively. The Kaplan-Meier curves in Supplementary Fig. 5c were plotted using survival (3.5-7) and survminer R package (0.4.9), and the p-value was calculated using log-rank test (Mantel-Cox test). The DAVID web server (https://david.ncifcrf.gov/) was used for GO enrichment analysis and the p-value was determined by a two-sided Fisher's exact test with Benjamini-Hochberg adjustment. KEGG enrichment analysis was performed using KOBAS web server (http://bioinfo.org/kobas/) and the p-value was tested by a two-sided Fisher's exact test with Benjamini-Hochberg adjustment. Venn diagram in Fig. 1b and Sankey dot plot in Fig. 1c were performed using the matplotlib python package (3.7.0). Statistical analyses of the remaining data were done by GraphPad Prism statistical software. Data are shown as mean ± SEM. Statistical significance is provided in the figure legends. Statistical significance of egg deposition and hatchability in Fig. 2 and Fig. 6 was determined using the Kruskal-Wallis test followed by Dunn's post-hoc tests with Benjamini-Hochberg adjustment for multiple comparisons. A two-sided Fisher's exact test was used to statistically analyze the ratio of females producing hatchable eggs to total mosquitoes. Statistical significance of the mRNA abundance of LAP1 in Fig. 3a were determined using the one-way ANOVA test followed by Tukey's post hoc tests with Benjamini-Hochberg adjustment for multiple comparisons. Statistical significance of the expression of LAP1 in Fig. 3d and hatchability in Fig. 5 was determined using the two-sided multiple Mann-Whitney test with Benjamini-Hochberg adjustment. The p-value of the length of the ovarian follicles in Fig. 3c was determined by two-sided Mann-Whitney test. For curve fitting, all error bars represent ± SEM (Fig. 5c, 5f, 6c and 6f), which is 95% CI to indicate that the fitted curves are in the range of all error bars.

For manuscripts utilizing custom algorithms or software that are central to the research but not yet described in published literature, software must be made available to editors and reviewers. We strongly encourage code deposition in a community repository (e.g. GitHub). See the Nature Portfolio guidelines for submitting code & software for further information.

Data

Policy information about availability of data

All manuscripts must include a data availability statement. This statement should provide the following information, where applicable:

- Accession codes, unique identifiers, or web links for publicly available datasets
- A description of any restrictions on data availability
- For clinical datasets or third party data, please ensure that the statement adheres to our policy

The mass spectrometry proteomics data generated in this study have been deposited in the ProteomeXchange Consortium (http://proteomecentral.proteomexchange.org) via the iProX partner repository 69, 70 under accession number PXD039869. All data, supplementary information, and Source Data files are available in this article. Source data are provided with this paper.

Research involving human participants, their data, or biological material

Policy information about studies with human participants or human data. See also policy information about sex, gender (identity/presentation), and sexual orientation and race, ethnicity and racism.

Reporting on sex and gender	N/A
Reporting on race, ethnicity, or other socially relevant groupings	N/A
Population characteristics	N/A
Recruitment	N/A
Ethics oversight	N/A

Note that full information on the approval of the study protocol must also be provided in the manuscript.

Field-specific reporting

Please select the one below that is the best fit for your research. If you are not sure, read the appropriate sections before making your selection.

☒ Life sciences ☐ Behavioural & social sciences ☐ Ecological, evolutionary & environmental sciences

For a reference copy of the document with all sections, see nature.com/documents/nr-reporting-summary-flat.pdf

Life sciences study design

All studies must disclose on these points even when the disclosure is negative.

Sample size	The sample size for each experiment was described in the figure legends and determined based on statistical analysis. Sample sizes were chosen based on previous experience to achieve statistical significance and reproducibility. For SWATH-MS, spermathecae from 2000 individual female mosquitoes at the virgin (unmated) at 72 h PE, mated at 72 h PE and mated at 5 days PBM stages were pooled for the construction of a spectral library. Samples from 500 individual females at the virgin, 72 h PE and 5 days PBM stages were examined using data-dependent acquisition (DIA) mass spectrometry. For the egg deposition and hatchability in Fig 2, the experiments were independently performed twice with 50 females per experiment. For the egg deposition and hatchability in Fig 5 and Fig 6, the experiments were

independently performed three times with 30 females per experiment. For qPCR, sample sizes were designed for 3. For viral infection, seventy female mosquitoes in LAP1-/-♂ × LAP1-/-♀, LAP1-/+♂ × LAP1-/+♀, and WT♂ × WT♀ groups were infected with ZIKV. For survival rate, the experiments were independently performed three times with 30 males per experiment.

Data exclusions	The mosquitoes that died before measurement were excluded from the analysis.
Replication	SWATH-MS was performed once with three biological replicates. For the egg deposition and hatchability in Fig 2, the experiments were independently performed twice with 50 females per experiment. For the egg deposition and hatchability in Fig 5 and Fig 6, the experiments were independently performed three times with 30 females per experiment. qPCR was performed three times with three biological replicates. Viral infection was performed three times with seventy female mosquitoes. Survival rate was performed three times with 30 biological samples. For immunofluorescence assay and transmission electron microscopy were repeated three times with similar results.
Randomization	Mosquitoes were randomly allocated to the experiments.
Blinding	There was no blinding in these studies. The experimental design did not require blinding because the variables assessed are not confounded by the assessor. We focused only on measurable variables (egg deposition, hatchability, viral titer, survival rate, etc.).

Reporting for specific materials, systems and methods

We require information from authors about some types of materials, experimental systems and methods used in many studies. Here, indicate whether each material, system or method listed is relevant to your study. If you are not sure if a list item applies to your research, read the appropriate section before selecting a response.

Materials & experimental systems

n/a	Involved in the study
☐	☒ Antibodies
☐	☒ Eukaryotic cell lines
☒	☐ Palaeontology and archaeology
☐	☒ Animals and other organisms
☒	☐ Clinical data
☒	☐ Dual use research of concern
☒	☐ Plants

Methods

n/a	Involved in the study
☒	☐ ChIP-seq
☒	☐ Flow cytometry
☒	☐ MRI-based neuroimaging

Antibodies

Antibodies used	For immunofluorescence assay, anti-LAP1 antibody (1:500) and Alexa Fluor 488-conjugated anti-mouse secondary antibody (1:2000) (Cat. A11001, Invitrogen™, USA) were used. Hoechst 33258 (Cat. H3569, Invitrogen™, USA) was used to stain the nucleus at a final concentration of 2 µg/mL. For Western blot, anti-LAP1 primary antibody (1:5000), conjugated anti-mouse IgG (H&L)-HRP secondary antibody (1:10000) (Cat. BE0102-100, EASYBIO, China), and β-actin mouse monoclonal antibody (1:5000) (Cat. BE0033-100, EASYBIO, China) were used.
Validation	Anti-LAP1 antibody: the LAP1 mouse polyclonal antibody was purified at Beijing Protein Innovation Co., Ltd. Western blot were performed to confirm its reactivity with Aedes aegypti LAP1 based on the predicated molecular weight of the protein. Data provided in the a Source Data file.<br>Alexa Fluor 488-conjugated anti-mouse secondary antibody: Antibody specificity was validated by ICC/IF on the website (https://www.thermofisher.cn/cn/zh/antibody/product/Goat-anti-Mouse-IgG-H-L-Cross-Adsorbed-Secondary-Antibody-Polyclonal/A-11001).<br>Hoechst 33258: Hoechst 33258 was validated by ICC/IF on the website (https://www.thermofisher.cn/order/catalog/product/H3569?SID=srch-srp-H3569).<br>Conjugated anti-mouse IgG (H&L)-HRP secondary antibody: Antibody specificity was validated by WB on the website (http://bioeasytech.com/product/2907.html?goods_id=5794).<br>Mouse monoclonal β-actin antibody: Antibody specificity was validated by WB on the website (http://bioeasytech.com/product/2379.html?goods_id=4297).

Eukaryotic cell lines

Policy information about cell lines and Sex and Gender in Research

Cell line source(s)	C6/36 cells (ATCC, CRL-1660)
Authentication	None of the cell lines used were authenticated.
Mycoplasma contamination	C6/36 cells were not tested for mycoplasma contamination.
Commonly misidentified lines (See ICLAC register)	None.

Animals and other research organisms

Policy information about studies involving animals; ARRIVE guidelines recommended for reporting animal research, and Sex and Gender in Research

Laboratory animals	Liverpool strain Ae. aegypti mosquitoes were reared by supplementation with 10% (wt/vol) sucrose solution and water. The larval mosquitoes were fed with the mixture of powdered mouse food, albumin and yeast extract. The rearing conditions were 28?, 80% humidity with a 12/12-hour light/dark cycle. Mosquito strains were maintained by feeding with chicken (4 to 8 weeks old) blood once a month.
Wild animals	No wild animals.
Reporting on sex	Mosquito experiments includes males and females.
Field-collected samples	No field-collected samples.
Ethics oversight	All mosquito experiments were performed strictly following bioethics principles and were supervised by the Bioethics Committee of the Institute of Zoology, Chinese Academy of Science.

Note that full information on the approval of the study protocol must also be provided in the manuscript.
